# IgG4-related Disease Manifesting as Autoimmune Pancreatitis, Sialadenitis, and Cholangitis: Case Report

**DOI:** 10.15388/Amed.2025.32.2.11

**Published:** 2025-12-30

**Authors:** Augustina Tumelytė, Vytautas Rimkus, Paulina Tekoriutė, Limas Kupčinskas

**Affiliations:** 1 Faculty of Medicine, Academy of Medicine, Lithuanian University of Health Sciences, Kaunas, Lithuania; 2 Faculty of Medicine, Academy of Medicine, Lithuanian University of Health Sciences, Kaunas, Lithuania; 3 Department of Gastroenterology, Faculty of Medicine, Academy of Medicine, Lithuanian University of Health Sciences, Kaunas, Lithuania; 4 Department of Gastroenterology, Faculty of Medicine, Academy of Medicine, Lithuanian University of Health Sciences, Kaunas, Lithuania; European Reference Network on Hepatological Diseases (ERN RARE-LIVER), Hamburg, Germany

**Keywords:** IgG4-related disease, autoimmune pancreatitis, sialadenitis, cholangitis, su IgG4 susijusi liga, autoimuninis pankreatitas, sialoadenitas, cholangitas

## Abstract

IgG4-related disease (IgG4-RD) is a rare autoimmune disease which can affect almost any organ. We present a case of IgG4-RD, which manifested as chronic autoimmune pancreatitis with pancreatic insufficiency, severe malnutrition, autoimmune sialadenitis, and cholangitis. After the administration of prednisolone and mycophenolate mofetil, the patient’s condition improved significantly.

## Abstract (it)

Su IgG4 susijusi liga (angl. *IgG4-Related Disease*) yra reta autoimuninė liga, kuri gali pažeisti beveik kiekvieną organą. Pristatome šios ligos atvejį, kai šia liga sergant įtariamai pacientei buvo lėtinis autoimuninis pankreatitas ir kasos nepakankamumas, sunkus mitybos nepakankamumas, autoimuninis sialoadenitas ir cholangitas. Po skirto gydymo prednizolonu ir mikofenolato mofetilu pacientės būklė kur kas pagerėjo.

## Introduction

IgG4-related disease (IgG4-RD) can manifest in nearly any organ, with frequent manifestations in major salivary and lacrimal glands, orbits, pancreas, retroperitoneum, as well as renal tubules, and interstitium [1]. This rare autoimmune disease affected 1.39 per 100,000 people in 2019, according to a study completed with the United States of America population [2]. The main diagnostic indicator of IgG4-RD is increased serum IgG4 levels, with 84% of patients having above 1.35 g/L [3]. Nonetheless, the exact role of IgG4 in the immunopathogenesis of this disease remains unclear. It has been observed that the fibrotic subtype of IgG4-RD can present with normal IgG4 serum levels. Tissue infiltration by IgG4+ plasma cells is also a distinctive trait of IgG4-RD [4]. Although not fully understood, the pathogenesis is of immune-mediated origin. The absence of infectious triggers, the clinical presentation, and the usually adequate response to glucocorticoids (GC) or other immunosuppressants support this theory [1]. Both the peripheral blood and fibrotic lesions of IgG4-RD patients contain an increased amount of CD4+ cytotoxic T lymphocytes [5]. Moreover, individuals with IgG4-RD have higher levels of plasmablasts and plasma cells, which are associated with an increased disease activity [6,7]. Recognized imaging patterns can aid in diagnosis; however, general imaging findings may be nonspecific, making it difficult to distinguish IgG4-RD from other inflammatory or oncologic conditions reliably [8]. GC are considered the first-line therapy for IgG4-RD [9]. To avoid long-term GC adverse effects, alternative immunosuppressive agents can be introduced to sustain remission [1].

We present a clinical case from Lithuania of IgG4-RD manifesting in a 41-year-old female as autoimmune pancreatitis, sialadenitis, and IgG4-related cholangitis.

## Case Presentation

We present a 41-year-old female hospitalized in the Gastroenterology Department of Kaunas Clinics with complaints of severe generalized pruritus, hypertrophy and tenderness of the salivary glands, pain under the left rib cage, and white-colored diarrhea. At the age of 25, the patient was diagnosed with chronic idiopathic pancreatitis with exocrine and endocrine insufficiency. The anamnesis revealed marked weight loss (3 kg/month), exacerbation of osteoporosis, and increasingly recurring sialadenitis. To investigate the cause of severe malnutrition, a psychiatric assessment was performed; however, no eating disorder was confirmed, and referrals for nutritional, osteoporosis, and psychiatric management were made. At the time of hospitalization, the patient was taking fluoxetine, zolpidem, pancreatin, omeprazole, ursodeoxycholic acid, nonsteroidal anti-inflammatory drugs, and specialized nutritional supplements.

**Fig. 1:**
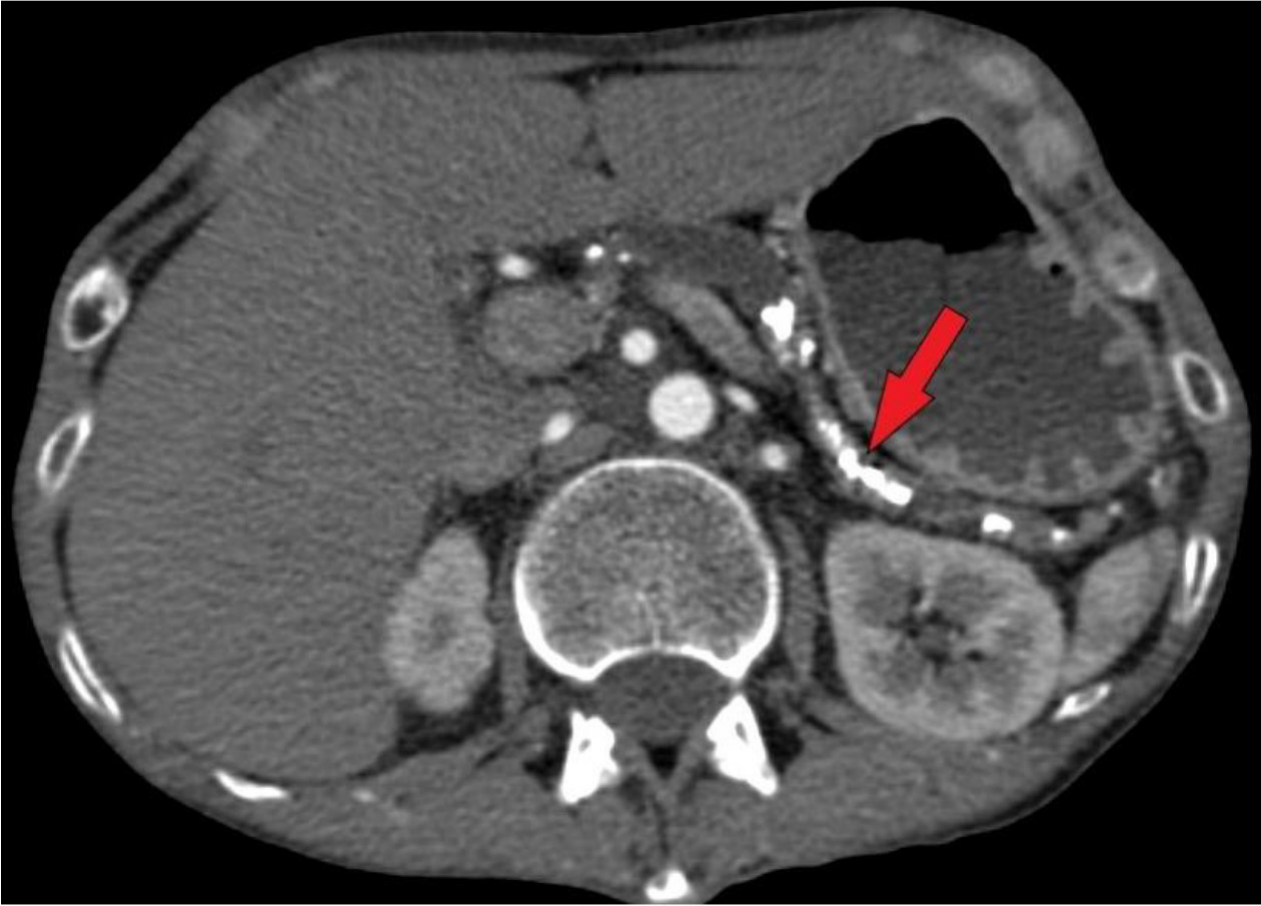
Axial plane of contrast-enhanced abdominal and pelvic CT demonstrates multiple pancreatic calcifications (red arrow), along with dilation of the pancreatic duct measuring up to 0.8 cm. These findings are consistent with chronic pancreatitis. Image courtesy of the Radiology Department, Kaunas Clinics

Physical examination revealed bilateral parotid hypertrophy, restricted jaw movement, and abdominal tenderness in both upper quadrants. The patient’s body mass index (BMI) was only 11.97 kg/m^2^ (Height = 171 cm; Weight = 35 kg). The psychiatrists suspected bulimia nervosa and anorexia nervosa, manifesting along with chronic idiopathic pancreatitis with pancreatic insufficiency. That was because of the patient’s tremendously low BMI, which is frequently seen in eating disorders. Parotid gland enlargement and tenderness also supported this theory because it resembled a typical ‘bulimia face’ appearing with ‘chipmunk-like’ sialadenosis. However, the patient had no history of eating disorders or binge eating as confirmed before. Laboratory tests showed hypokalemia, elevated α-amylase concentration of 385 U/L (cf. normal: 28–100 U/L), normal pancreatic amylase levels, and an increase of bile acids (20 µmol/L). Although in ultrasound (US) and computed tomography (CT) imaging no significant damage in the bile ducts was seen, the bile acid elevation and marked itchiness indicated cholangitis. Abdominal US revealed a heterogeneous pancreas with parenchymal calcifications and a dilated pancreatic duct (0.9 cm wide), containing a 0.5 cm intraductal stone in the pancreatic head. US showed a heterogeneous parotid gland structure, with the right parotid gland measuring 5.2 × 2.9 cm and the left 4.9 × 2.8 cm. Abdominal and pelvic CT imaging revealed multiple pancreatic calcifications, up to 0.8 cm dilated pancreatic duct, consistent with chronic pancreatitis (Fig. 1). A review of previous laboratory findings indicated that elevated IgG4 levels (up to 2 g/L) had been present since 2018.

The patient was diagnosed with IgG4-RD, including its manifestations: chronic autoimmune pancreatitis (AIP), autoimmune sialadenitis, and early-stage IgG4-related cholangitis, which could not be excluded as a possible manifestation due to elevated bile acids and severe, persistent whole-body pruritus. Treatment was initiated with calcium (1200 mg) and vitamin D (800 IU) supplementation, along with a short course of GC at a target dose of 0.6 mg/kg for 4 weeks. Due to concerns that GC therapy might worsen osteoporosis, the patient was transferred to the Endocrinology Department. The vitamin D dose was increased to 6000 IU/day for 3 months, and a dual-energy X-ray absorptiometry (DEXA) scan confirmed persistent osteoporosis. US revealed heterogeneous, enlarged salivary glands with a few isolated hypertrophic lymph nodes, prompting a biopsy of the salivary glands. However, due to an insufficient amount of tissue obtained during the biopsy, an immunohistochemistry test for IgG4-RD could not be performed. The patient was started on prednisolone (0.6 mg/kg) and azathioprine (50 mg/day) maintenance therapy; however, due to poor tolerance, it was replaced with mycophenolate mofetil (250 mg/day).

Osteoporosis treatment was revised and continued with denosumab (60 mg/ml every 6 months). A clinical dietitian managed the patient’s malnutrition. Aside from electrolyte correction, adequate IV fluid therapy, analgesics, and spasmolytics were administered, while proton pump inhibitor and pancreatin were taken with main meals. Eventually, the patient’s condition improved as pruritus resolved, IgG4 concentration decreased to 0.7 g/L, bile acid levels reached 4 µmol/L, salivary glands returned to the normal size, and the patient’s weight increased by 4 kg.

## Discussion

IgG4-RD usually occurs without an abrupt onset of general symptoms such as fever. Only a fraction of IgG4-RD patients experience weight loss and have marked elevations in acute-phase markers, along with other signs of systemic inflammation [5]. Our patient experienced weight loss and was severely underweight.

Low BMI and parotid gland enlargement are harmful consequences of repeated vomiting, usually a result of dysfunctional eating habits. Bulimia nervosa and anorexia nervosa can manifest in extreme food restriction or binge eating episodes [10]. Although our patient’s physical appearance was indicative of a possible eating disorder, this theory was excluded after a psychiatric assessment.

Type 1 AIP is more likely to affect elderly males and has a lower likelihood of causing jaundice and abdominal pain [11]. However, our patient reported pain under the left rib cage. The majority of type 1 AIP patients experience extrapancreatic IgG4-RD manifestations [11], which was also observed in this case.

Pancreatic imaging typically reveals generalized or segmental pancreatic enlargement, coupled with the loss of normal lobularity and diffuse stenosis of the pancreatic duct [1]. Our patient’s abdominal US and CT revealed dilation of the pancreatic duct due to intraductal concrements.

Type 1 AIP is often linked to IgG4-related cholangitis, which manifests in 20% of patients with multiorgan involvement [1], as seen in our case.

Salivary and lacrimal glands are also involved in almost 40 percent of type 1 AIP patients [12]. In IgG4-related sialadenitis (IgG4-RS), the first symptom is typically bilateral swelling of the submandibular or lacrimal glands [13]. However, our patient had parotid gland enlargement. Besides the parotid glands, sublingual and labial salivary glands can also be involved. For this reason, US imaging is the most commonly used diagnostic tool, as it is non-invasive and cost-effective [5]. In our patient’s case, US confirmed glandular involvement.

Positron emission tomography/ computed tomography (PET/CT) is considered a valuable tool for detecting affected areas in multifocal disease and for identifying safe biopsy sites [14]. However, this method was not utilized in this particular case.

GC treatment should be initiated at a dose of 0.6–0.8 mg/kg body weight per day orally for 1 month to induce remission, followed by an evaluation of the response to treatment [15]. Maintenance GC therapy is recommended for patients with high disease activity or an increased risk of recurrence [9]. In our case, the patient did not require GC maintenance therapy and was started on immunosuppressive therapy with azathioprine, which was later replaced with mycophenolate mofetil due to poor tolerance.

When administered every 6 months, anti-CD20 monoclonal antibody rituximab (RTX) is an effective treatment for maintaining IgG4-RD remission [16]. RTX is generally well-tolerated and considered safe for IgG4-RD patients [17]. However, in this case, RTX was not required, as the patient showed an adequate response to mycophenolate mofetil.

The diagnosis of IgG4-RD is based on the 2020 revised comprehensive diagnostic criteria by Umehara et al., which include:

- Clinical and radiological characteristics suggestive of IgG4-RD
- Serum IgG4 levels >1.35 g/L
- At least two histopathological findings indicative of IgG4-RD, such as immune cell infiltration with fibrosis, storiform fibrosis, obliterative phlebitis, or ratio of IgG4-positive plasma cells and IgG-positive cells >0.40, and the number of IgG4-positive plasma cells >10 per high-power field [18].

However, there are studies stating that this disease can be diagnosed despite the absence of histological findings, when there are clinical and radiological features suggestive of IgG4-RD, elevated IgG4 concentration, and responsiveness to immunosuppressants [19–22]. This was the case for our patient, as she had significantly elevated serum IgG4 levels (2 g/L), as well as clinical and radiological signs characteristic of IgG4-RD. Although the inflammatory signs were non-specific, an effective response to immunosuppressants confirmed the clinical diagnosis of IgG4-RD. Nevertheless, the absence of histological confirmation remains a limitation of this case report, since it would have aided in solidifying this diagnosis even further.

## Conclusions

The rarity and diverse manifestations of IgG4-RD complicate the diagnostic process for clinicians. However, typical multiorgan involvement, elevated IgG4 concentration, radiological features, and favorable response to immunosuppressants make this process possible even if the histological confirmation is absent. GC, along with other immunosuppressive agents, is the primary treatment option for this disease. This case demonstrates delayed IgG4-RD diagnosis, complicated by pancreatic insufficiency. This proves that early diagnostics of IgG4-RD is essential and can ensure better outcomes.
